# Chronic Activation of the Renin-Angiotensin System Induces Lung Fibrosis

**DOI:** 10.1038/srep15561

**Published:** 2015-10-23

**Authors:** Jiaolong Wang, Li Chen, Bohao Chen, Angelo Meliton, Shu Q. Liu, Yongyan Shi, Tianjing Liu, Dilip K. Deb, Julian Solway, Yan Chun Li

**Affiliations:** 1 Department of Medicine, Division of Biological Sciences, The University of Chicago, Chicago, IL 60637, USA; 2 Division of Cardiology, The First Affiliated Hospital, China Medical University, Shenyang, Liaoning, 110001, China; 3 Division of Endocrinology, Shengjing Hospital, China Medical University, Shenyang, Liaoning, 110004, China; 4 Department of Biomedical Engineering, Northwestern University, Evanston, IL 60208, USA; 5 Division of Neonatology, Shengjing Hospital, China Medical University, Shenyang, Liaoning, 110004, China

## Abstract

Pulmonary fibrosis is a serious lung disorder that can lead to respiratory failure. Here we show that transgenic mice expressing active renin from the liver (RenTgMK) developed progressive pulmonary fibrosis leading to impaired pulmonary function. Histological analyses revealed a marked increase in extracellular matrix (ECM) deposition and decrease in alveolar size in the lungs of RenTgMK mice compared to wild-type (WT) littermates, accompanied with increased expression of ECM proteins and fibrogenic factors. The increase in lung fibrosis led to a substantial decrease in respiratory system compliance. Two-week treatment with aliskiren (renin inhibitor) or losartan (AT1 antagonist) ameliorated pulmonary ECM deposition, blocked the induction of ECM proteins and fibrogenic factors and improved respiratory compliance in RenTgMK mice, confirming a critical role of the renin-Ang II-AT1 cascade in promoting pulmonary fibrogenesis. However, when RenTgMK mice were treated with hydralazine (a smooth muscle relaxant), the blood pressure was normalized but the lung fibrotic abnormalities, fibrogenic gene induction and pulmonary elasticity were not corrected. Moreover, intratracheal instillation of lipopolysaccharide induced more severe lung injury in RenTgMK mice compared to WT littermates. These observations demonstrate that the renin-angiotensin system is a key mediator of lung fibrosis, and its pro-fibrotic effect is independent of blood pressure.

Pulmonary fibrosis is a devastating lung problem manifested by excessive deposition of extracellular matrix (ECM) in the lung. Fibrotic lesions distort lung architecture and alveolar structure, thicken alveolar walls, reduce lung compliance, decrease oxygen diffusion capacity and ultimately impair lung function^1^. It is believed that lung fibrosis is a dysregulated wound-healing response following lung injury, induced by various causes including allergens, chemicals, radiation and environmental particles. Leukocyte recruitment following lung injury triggers pulmonary inflammation that releases cytokines, chemokines and growth factors. Excessive production of some of these factors, such as pro-fibrotic IL-13 and transforming growth factor (TGF)-β, promotes epithelial-to-mesenchymal transformation and stimulates interstitial fibroblast and myofibroblast proliferation and ECM production by these cells, leading to lung fibrosis^2^,^3^. Despite advances in this research area, however, the molecular basis of pulmonary fibrosis remains incompletely understood.

One important pathogenic factor that has been implicated in the pathogenesis of lung fibrosis is the renin-angiotensin system (RAS)^4^. The RAS is a regulatory cascade that plays an essential role in the regulation of blood pressure, electrolyte and volume homeostasis. The first and rate-limiting component of this cascade is renin, an aspartyl protease that cleaves angiotensinogen to angiotensin (Ang) I, which is further converted to Ang II by angiotensin-converting enzyme (ACE). Ang II is the central effector of the RAS and exerts biological activities through binding to its receptors, AT1 and AT2^5^. In addition to the classic cardiovascular effects, Ang II has a range of other activities including proliferative and pro-fibrotic actions on fibroblasts, which are mediated mainly by the AT1 receptor^4^. In fact, the lung contains a high level of ACE, and increased ACE concentration has been reported in the broncho-alveolar fluid in fibrotic lung diseases^6^. Moreover, angiotensinogen is found to be one of the most overexpressed genes in patients with pulmonary fibrosis^7^. Together these probably contribute to an increase in Ang II production during lung injury and in fibrotic lungs. Ang II stimulates the expression of (TGF)-β and connective tissue growth factor (CTGF), two potent pro-fibrotic factors that drive lung fibroblast/myofibroblast proliferation and ECM protein expression^8^,^9^,^10^,^11^. In addition, inhibition of the RAS by ACE inhibitors or AT1 receptor blockers has been shown to ameliorate lung fibrosis in a number of experimental lung fibrosis models^12^,^13^,^14^. Although these studies have suggested a role of the RAS in lung fibrogenesis, generally speaking the evidence based on these and other studies is mostly circumstantial. Direct *in vivo* studies designed to test this concept remains scarce in the literature. In this report, we show that transgenic mice with chronic activation of the RAS spontaneously develop progressive lung fibrosis independently of blood pressure, and this abnormality results in a marked decline in pulmonary function. We believe that this work provides more compelling and more direct evidence to support a pro-fibrotic role of the RAS in the pathogenesis of pulmonary fibrosis.

## Results

### RenTgMK transgenic mice develop progressive lung fibrosis

**Figure 1:**
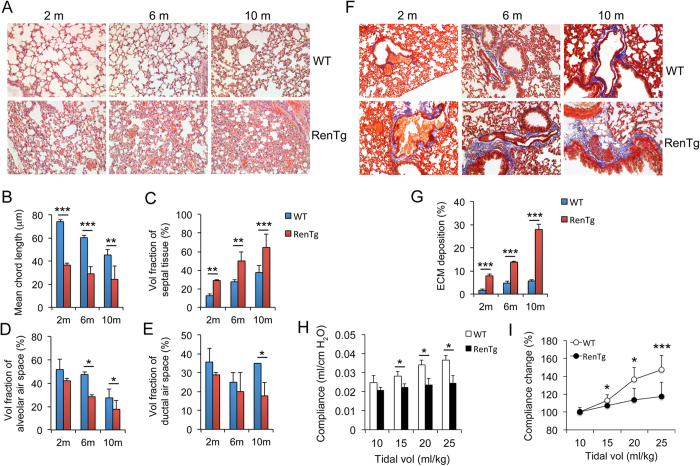
RenTgMK mice develop progressive lung fibrosis. RenTgMK and WT littermates were analyzed at 2, 6 and 10 months of age as indicated. (**A**) H&E staining of lung structure. Magnification 200x; (**B**) Mean chord length; (**C**) Volume fraction of septal tissue area; (**D**) Volume fraction of alveolar air space; (**E**) Volume fraction of ductal air space; (**F**) Masson’s trichrome staining. Magnification 200x; (**G**) ECM deposition area (%) estimated from Masson’s trichrome staining; (**H**) Dynamic respiratory system compliance during tidal ventilation at increasing tidal volumes at 2 months of age; (**I**) Respiratory system compliance at 2 months of age vs. tidal volume, normalized to the value at 10 ml/kg in percentage calculation. *P < 0.05; **P < 0.01; ***P < 0.001. n = 4–5 mice in each group. Vol, volume; m, month.

RenTgMK mice carry a single copy of the synthetic mouse renin cDNA driven by the albumin promoter/enhancer. This transgene was inserted to the apolipoprotein locus. Active renin protein is produced and secreted from the liver, and the transgenic mice have increased circulating levels of Ang II and develop cardiac hypertrophy and hypertension^15^. Cardiac fibrosis and renal interstitial fibrosis were previously reported in this transgenic mouse line^15^,^16^. In this study we found that RenTgMK mice developed lung fibrosis, the severity of which progressed with age. At two months of age, RenTgMK mice already showed irregular, distorted and collapsed alveolar structure and increased septal spaces, and these abnormalities became more severe at 6 and 10 months of age (Fig. 1A). Quantitatively, the mean chord length, which is the mean linear intercept length that measures the mean free distance in the air spaces and reflects alveolar size^17^, was substantially reduced in RenTgMK mice (Fig. 1B), whereas the fraction of septal tissue area was markedly increased in all age groups (Fig. 1C). At 10 months of age, the volume fraction of alveolar and ductal air spaces was also significantly decreased in the transgenic mice (Fig. 1D,E). Masson’s trichrome staining showed a substantial increase in ECM deposition in interstitial spaces surrounding bronchioles and blood vessels in RegTgMK mice, which was clearly progressive with aging (Fig. 1F,G). At 10 months of age, close to 30% of the lung tissue was deposited with ECM (Fig. 1G). Consequently, respiratory system compliance was markedly reduced in RenTgMK mice in comparison with WT littermates at as early as two months of age (Fig. 1H,I), demonstrating impaired pulmonary function in the transgenic mice.

**Figure 2:**
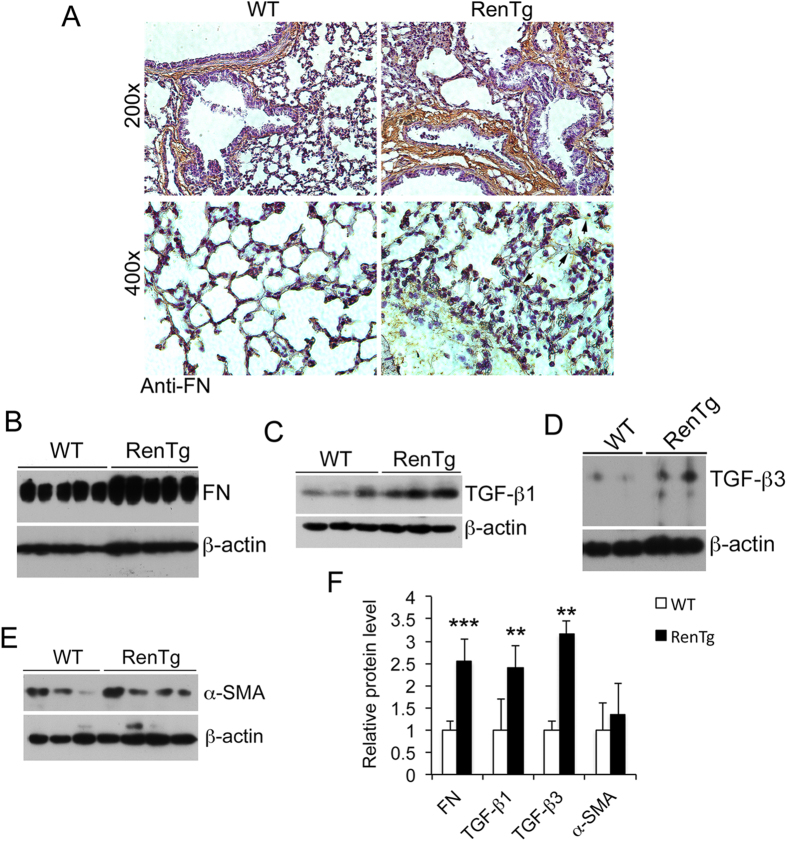
Increased production of extracellular matrix protein and pro-fibrotic factors in RenTgMK mice. (**A**) Lung immunostaining with anti-fibronectin antibody. Magnification 200x upper panels and 400x lower panels; (**B**–**E**) Western blot analysis of fibronectin (**B**), TGF-β1 (**C**), TGF-β3 (**D**) and α-SMA (**E**). (**F**) Densitometric quantitation of these proteins. **P < 0.01; ***P < 0.001 vs. WT. All gels were run under the same experimental condition, and gel images are cropped for concise presentation. All uncut gel images are provided in Supplementary Information.

To explore the development of lung fibrosis at molecular levels, we assessed the expression of extracellular matrix (ECM) proteins and pro-fibrotic growth factors in the lungs of 2-month old RenTgMK mice and WT controls. Immunostaining showed a marked increase in fibronectin deposition in RenTgMK lungs. Increased fibronectin deposition was easily detected in the space surrounding bronchioles and small blood vessels, and close examination at high magnification also revealed fibronectin deposition on the alveolar walls (Fig. 2A, see 400x panels), which may represent the accumulation of intra-alveolar fibroblasts or myofibrobalsts. Western blot analyses demonstrated >2.5- fold increase in fibronectin expression in the transgenic mice over WT controls (Fig. 2B,F). Moreover, lung lysates prepared from RenTgMK mice contained significantly higher concentrations of TGF-β1 and TGF-β3, well known pro-fibrotic factors, compared to those prepared from WT littermates (Fig. 2C,D and F). The level of α-SMA also trended higher in the transgenic mice, although the difference was not statistically significant (Fig. 2E,F).

### Lung fibrosis is induced by renin activity but independent of high blood pressure

**Figure 3:**
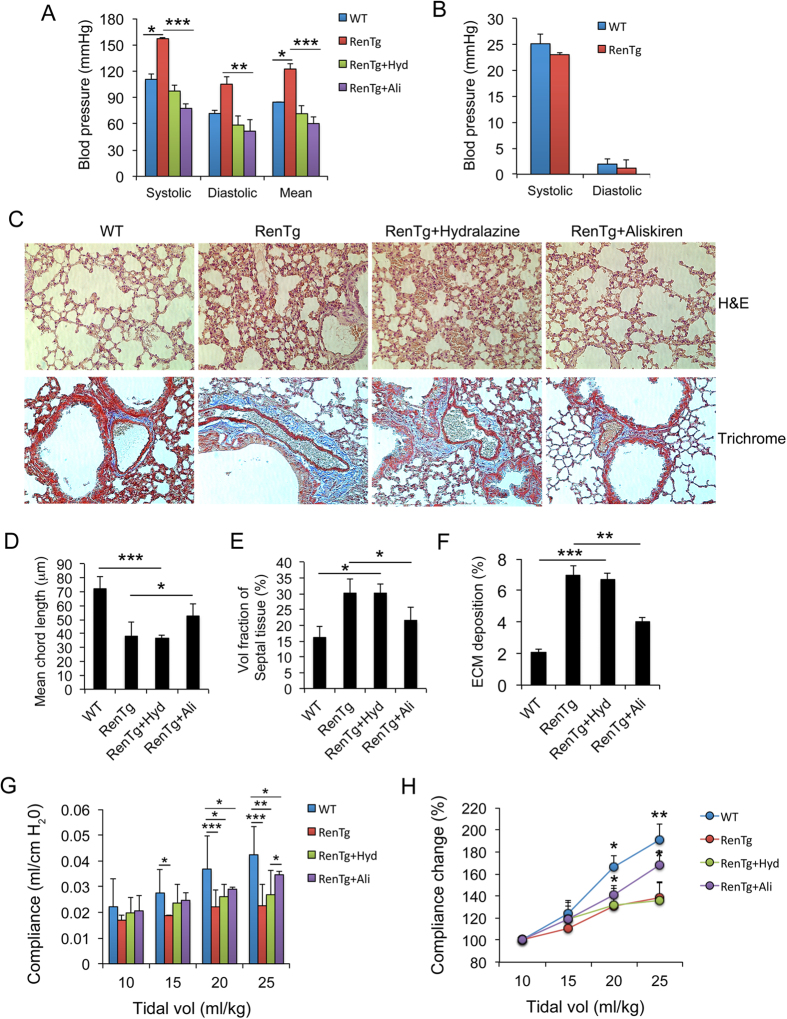
Lung fibrosis is blocked by renin inhibition but not by blood pressure reduction. RenTgMK mice were treated with hydralazine (20 mg/kg) or aliskiren (20 mg/kg) for two weeks. WT and RenTgMK controls were treated with PBS. (**A**) Systolic, diastolic and mean aortic blood pressure in these four groups of mice. *P < 0.05, **P < 0.01, ***P < 0.001. (**B**) Systolic and diastolic right ventricular blood pressure in WT and RenTgMK mice. (**C**) H&E staining (*upper panels*) and Masson’s trichrome staining (*lower panels*) of lung structure in these four groups of mice. Representative images are shown. These images are not sequential slices. (**D**) Mean chord length; (**E**) Volume fraction of septal tissue space; (**F**) ECM deposition area (%) estimated from Masson’s trichrome staining; (**G**) Respiratory system compliance during tidal ventilation at increasing tidal volumes; *P < 0.05, **P < 0.01, ***P < 0.001; (**H**) Normalized dynamic respiratory system compliance vs. tidal volume, as in Fig. 1I; *P < 0.05, **P < 0.01 vs. RenTg or RenTg + Hyd. n = 5–6 mice in each group.

**Figure 4:**
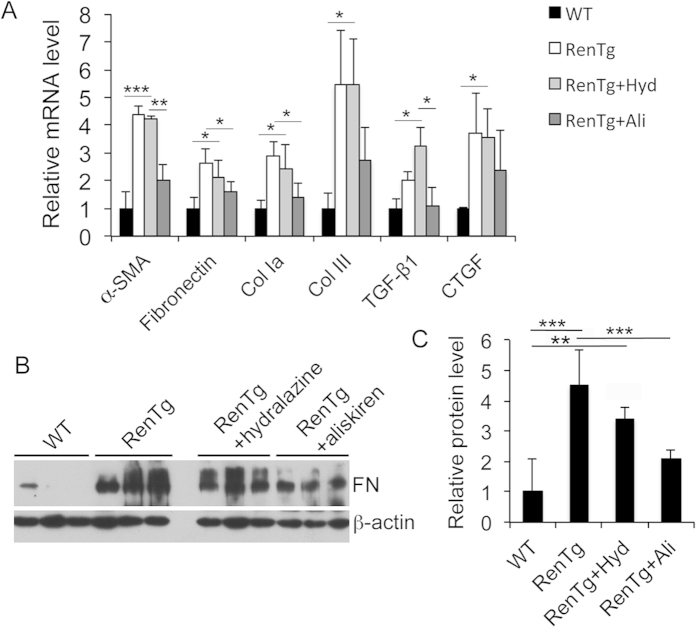
Effects of aliskiren and hydralazine on the expression of ECM proteins and pro-fibrotic factors. (**A**) Real time PCR quantitation of ECM proteins and pro-fibrotic factors; *P < 0.05, **P < 0.01, ***P < 0.001. (**B**,**C**) Western blot analysis and densitometric quantitation of fibronectin; **P < 0.01, ***P < 0.001. All gels were run under the same experimental condition, and gel images are cropped for concise presentation.

RenTgMK mice develop hypertension because of high renin activity^15^. Indeed, we confirmed that both systolic and diastolic blood pressure, measured from the carotid artery, were substantially elevated in RenTgMK mice (Fig. 3A), but right ventricular blood pressure, systolic or diastolic, which reflects pulmonary blood pressure, was not significantly different between WT and transgenic mice (Fig. 3B). Therefore, it is unlikely that pulmonary blood pressure is the cause of pulmonary fibrosis in RenTgMK mice, but the effect of high artery blood pressure and high renin activity on the development of pulmonary fibrosis needs to be addressed. To this end, we treated the transgenic mice with aliskiren, a specific renin inhibitor^18^, or with hydralazine, a smooth muscle relaxant anti-hypertensive drug that was previously shown to normalize blood pressure in RenTgMK mice^16^. Although both aliskiren and hydralazine were able to completely normalize systolic and diastolic blood pressure in RenTgMK mice (Fig. 3A), these two drugs had very different effects on lung fibrosis. Aliskiren mostly normalized the architecture of the RenTgMK lung (Fig. 3C), substantially increased the mean chord length (Fig. 3D), markedly reduced the septal tissue area in the RenTgMK lung (Fig. 3E), and substantially brought down ECM deposition in RenTgMK mice (Fig. 3C,F). In marked contrast, hydralazine treatment had little effect on the alveolar structure, chord length, septal tissue volume fraction and ECM deposition in the RenTgMK lung (Fig. 3C–F). As a result, aliskiren substantially improved respiratory system compliance of RenTgMK mice, but hydralazine had little effect on this lung function parameter (Fig. 3G,H). At the molecular level, quantitative RT-PCR confirmed that aliskiren significantly abrogated the induction of pro-fibrotic factors (TGF-β1 and CTGF), myofibroblast marker (α-SMA) and ECM proteins (fibronectin, collagen I and III) in the transgenic mice, but hydralazine had no effect on these genes (Fig. 4A). Consistently, aliskiren, but not hydralazine, dramatically blocked the induction of fibronectin (Fig. 4B,C) in RenTgMK mice. Taken together, these results indicate that the development of pulmonary fibrosis is mainly attributed to the high renin activity in the transgenic mice, but is independent of the high blood pressure.

### Lung fibrosis is induced by Ang II-AT1 receptor signaling

**Figure 5:**
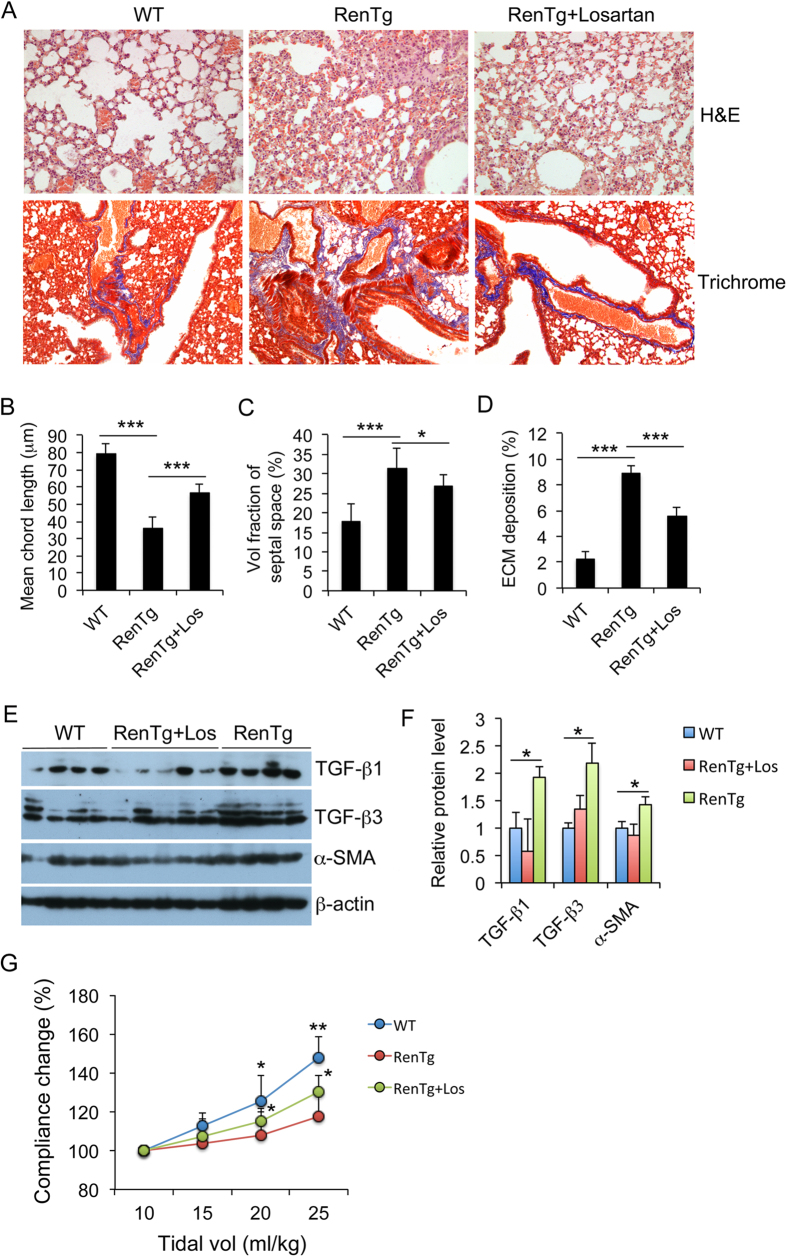
Lung fibrosis is blocked by inhibition of AT1 receptor signaling. RenTgMK and WT littermates were treated with losartan for two weeks before sacrifice for lung analysis. (**A**) H&E staining (*upper panels*) and Masson’s trichrome staining (*lower panels*). Magnification 200x. Representative images are shown. These images are not sequential slices. (**B**) Mean chord length; (**C**) Volume fraction of septal area (%); (**D**) ECM deposition (%); *P < 0.05; ***P < 0.001. (**E**,**F**) Western blot analysis of TGF-β1, TGF-β3 and α-SMA (**E**) and densitometry quantitation (**F**). *P < 0.05; n = 4–5. All gels were run under the same experimental condition, and gel images are cropped for concise presentation. (**G**) Normalized dynamic respiratory system compliance vs. tidal volume, as in Fig. 1I. *P < 0.05, **P < 0.01 vs. RenTg; n = 4–5 in each group.

RenTgMK mice have a high level of circulating Ang II^15^. Although the pro-fibrotic activity of Ang II is well established, there are studies in the literature that suggest renin itself can directly cause lung fibrosis independently of Ang II^19^. To address whether the pulmonary fibrosis seen in RenTgMK mice is induced by the Ang II-AT1 signaling pathway, we pretreated WT and RenTgMK littermates with losartan, an AT1 antagonist, for 2 weeks before performing lung analyses. As shown in Fig. 5, losartan effectively improved alveolar structure and attenuated ECM deposition in the lung of RenTgMK mice (Fig. 5A). Quantitative assessment confirmed that losartan partially normalized the mean chord length and septal area (Fig. 5B,C) and substantially reduced ECM deposition (Fig. 5D) in the transgenic mice. At the molecular level, losartan treatment significantly blocked the induction of TGF-β1, TGF-β3 and α-SMA in the transgenic mouse lung (Fig. 5E,F). Finally, losartan treatment partially reversed the decline in the respiratory system compliance in RenTgMK mice (Fig. 5G). Taken together, these data confirmed that the lung fibrogenesis in the transgenic mice is caused, at least in part, by activation of the Ang II-AT1 receptor signaling pathway.

### RenTgMK mice developed more severe acute lung injury in response to LPS

**Figure 6:**
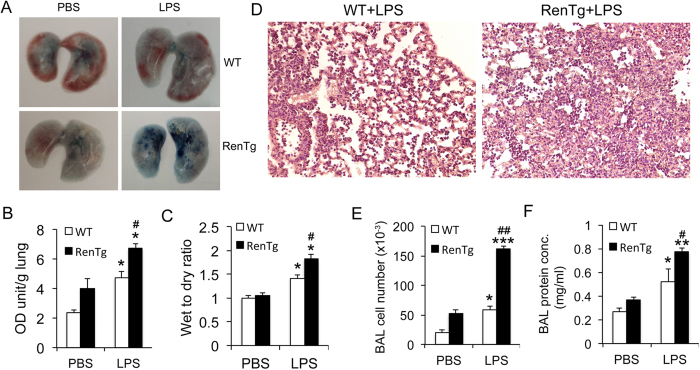
RenTgMK mice are more susceptible to LPS-induced acute lung injury. RenTgMK mice and WT littermates were treated with PBS or LPS (20 mg/kg) intratracheally. Lung injury analyses were performed 24 hours after LPS treatment. (**A**) Lung appearance after Evans blue dye injection; (**B**) Evans blue permeability assay. OD, optical density; (**C**) Wet to dry lung weight ratio; (**D**) H&E stained lung histology from mice treated with LPS; (**E**) Cell number in broncho-alveolar lavage (BAL) fluid; (**F**) Protein concentration in BAL fluid. *P < 0.05, **P < 0.01, ***P < 0.001 vs. PBS-treated in the same group; ^#^P < 0.05, ^##^P < 0.01 vs. WT of the same treatment. n = 4–6 mice in each group.

Lung fibrosis alters lung architecture and composition that can lead to increased susceptibility to lung injury. We used LPS-induced acute lung injury model to test this hypothesis. As showed in Fig. 6, intratracheal LPS distillation caused a more dramatic increase in pulmonary permeability in RenTgMK mice compared to WT mice, reflected by increased interstitial retention of Evans blue dye (Fig. 6A,B), as well as increased fluid retention in the lung, reflected by increased wet weight to dry weight ratio of the lung (Fig. 6C). Histological examinations confirmed that LPS induced more robust leukocyte infiltration in RenTgMK lung (Fig. 6D). Consistently, LPS challenge led to a greater increase in cell number and protein content in the BAL fluid in RenTgMK mice than in WT mice (Fig. 6E,F). Together these data demonstrate that LPS causes more severe acute lung injury in the transgenic mice.

## Discussion

The renin-angiotensin system has been suggested to play a role in the pathogenesis of lung fibrosis following lung injury. This speculation is mainly based on the observations that angiotensinogen and Ang II levels are increased in patients with lung fibrosis and relevant animal models^20^,^21^, on *in vitro* studies showing that Ang II or renin is able to induce lung fibroblast proliferation and ECM production, and on intervention studies showing that blockade of the RAS can ameliorate lung fibrosis. The unifying hypothesis is that Ang II production is increased following lung injury, as a result of increased angiotensinogen expression in damaged lung epithelial cells and activated myofibroblasts, to stimulate fibrogenesis^4^; however, there are in fact few studies in the literature that directly prove *in vivo* pro-fibrotic activities of the RAS in the context of lung fibrosis. The RenTgMK mice are genetic equivalent of chronic Ang II infusion by a minipump that is lifelong and noninvasive^15^. Thus, these mice provide an ideal model to assess the role of the RAS in lung fibrosis.

In this report we showed that these transgenic mice spontaneously develop progressive pulmonary fibrosis that impairs lung function. The development of lung fibrosis depends upon the renin-Ang II-AT1 cascade, but is independent of high blood pressure. We also demonstrated that the fibrotic abnormality renders the lung more susceptible to acute lung injury when exposed to LPS. Together our data provide compelling evidence that the RAS, particularly the activation of the Ang II-AT1 signaling pathway, plays a critical role in the pathogenesis of pulmonary fibrosis.

Previous studies reported that renal and cardiac fibrosis could be detected in 5-month old^22^ and 8–10-month old RenTgMK mice^15^,^16^. Here we showed that lung fibrosis develops much earlier in life (~2 months of age) in this transgenic model, which progresses with aging. By 2–6 month of age, lung fibrosis has already become very robust. Therefore, the lung appears to be more susceptible to the pro-fibrotic insult of chronic RAS activation than the kidney or the heart. At histological levels increased ECM deposition dramatically alters the lung architecture and distorts the alveolar structure in the transgenic mice, which exhibit a significant reduction in the mean chord length (i.e., the mean free distance in the air spaces) and a significant increase in the septal interstitial area. At old ages, alveolar and ductal air space were significantly reduced. Undoubtedly reflecting these structural abnormalities, respiratory system compliance was markedly lowered in the transgenic mice.

In RenTgMK mice the hepatic secretion of active renin elevates renin enzymatic activity and circulating Ang II levels independently of the body’s homeostatic control mechanisms, and these mice thus develop severe hypertension. One important question is whether the pulmonary fibrosis seen in these transgenic mice is a consequence of renin increase, activation of Ang II-AT1 signaling pathway or high blood pressure. This question needs to be addressed because (1) renin, through activating the prorenin/renin receptor, has been suggested to be a pro-fibrotic mediator in lung fibrosis independent of Ang II^19^; (2) both AT1 and AT2 receptors are thought to play a role in mediating lung fibrosis^21^,^23^; (3) high blood pressure is well known to have pathological effects on the renal, cardiovascular and pulmonary systems. In this study, by using aliskiren to directly inhibit renin enzymatic activity, we demonstrated that renin activity is required for the development of lung fibrosis in this model; by using hydralazine to normalize blood pressure in RenTgMK mice, which does not interfere with the RAS, we showed that the development of lung fibrosis is independent of high blood pressure; and by blocking the AT1 receptor signaling with losartan, we proved that lung fibrosis is at least in part induced by the activation of the AT1 receptor signaling pathway. Notably, neither aliskiren nor losartan completely reversed lung fibrosis and normalized the lung structure. This could be because two-week treatment may be insufficient to reverse the fibrotic abnormalities, though AT1-independent pro-fibrotic effects cannot be excluded. Nevertheless, these experimental data provide good evidence to conclude that activation of the renin-Ang II-AT1 cascade is largely responsible for pulmonary fibrosis in RenTgMK mice and the pro-fibrotic effect is independent of high blood pressure.

We also assessed the effect of lung fibrosis on acute lung injury. Intratracheal administration of LPS is known to cause lung injury by damaging the alveolar epithelial barrier, leading to infiltration of protein and cells from the blood to the broncho-alveolar space^24^. We showed that following LPS challenge, RenTgMK mice developed more robust lung injury than WT controls, indicating that the fibrotic lungs of RenTgMK mice are more susceptible to LPS-induced lung injury. It seems likely that this increased susceptibility is a direct consequence of lung fibrosis. However, direct effects of the renin-Ang II-AT1 cascade on lung injury independent of fibrosis cannot be excluded. In fact, Ang II and AT1 receptor have been reported to be up-regulated in the lung exposed to LPS, and inhibition of the Ang II-AT1 receptor signaling attenuates LPS-induced acute lung injury in the absence of lung fibrosis^25^,^26^. These studies suggest that Ang II induces pulmonary inflammation through the NF-κB and AP-1 pathways. Further investigation is warranted to address this issue.

Our data suggest that the molecular basis underlying the development of lung fibrosis in this transgenic model is Ang II stimulation of lung fibroblasts/myofibroblasts, directly or indirectly. In the lungs of RenTgMK mice ECM production, including fibronectin and collagen I and III, is greatly increased. It is known that these ECM proteins are mainly produced by activated fibroblasts or myofibroblasts. The expression of α-SMA, a myofibroblast marker, also tended to be greater in the lungs of RenTgMK mice. Moreover, these mice over-express TGF-β and CTGF, two potent pro-fibrotic growth factors known to stimulate fibroblast/myofibroblast proliferation and ECM protein production in a variety of tissues or organs including the liver, kidney, heart and lung^27^. Ang II, through activation of AT1 receptor, not only directly stimulates ECM protein production, but also induces TGF-β and CTGF expression from myofibroblasts^9^,^10^. Therefore, there are complex paracrine and autocrine networks operating in the lung to promote pulmonary fibrosis, and Ang II appears to act as an upstream driver of these networks in lung fibrogenesis.

Fibrotic lung disease is a devastating disorder that has no effective therapeutic treatment clinically^1^. Here we used a well-defined genetic experimental animal model to demonstrate that chronic activation of the RAS can indeed induce lung fibrosis. This conclusion provides a molecular basis for exploring anti-RAS therapy in the management of pulmonary fibrosis in human patients. Given the availability of a wide spectrum of anti-RAS drugs and their relatively well tolerance in long term use, future studies to explore their efficacy in lung fibrosis are warranted.

## Methods

### Animals and treatment

RenTgMK mouse, a transgenic mouse line that carries a single copy of mouse renin transgene driven by liver-specific albumin promoter/enhancer was described previously^15^. This transgenic line (on 129SV background) was purchased from Jackson laboratory (Stock # 007853) and a colony was established in our laboratory. Unless specifically indicated, RenTgMK mice and wild-type (WT) littermates were routinely sacrificed at two, six and ten months of age for lung analyses. In some experiments, two-month old RenTgMK mice and WT littermates were treated with aliskiren, hydralazine, or losartan for two weeks before sacrifice for analysis. Both aliskiren and hydralazine treatments were carried out by daily intraperitoneal injection at 20 mg/kg/day (both dissolved in PBS), and these doses were selected based on previous publications^16^,^28^. Losartan was placed in drinking water to give a treatment dose of 10 mg/kg/day. All animal experimental protocols described in this study were approved by the Institutional Animal Care and Use Committee of The University of Chicago. All animal experiments were carried out in accordance to Guide for the Care and Use of Laboratory Animals From National Research Council, Washington D.C.

### Measurement of blood pressure

Mouse artery blood pressure was determined by the carotid artery cannulation method as described previously^29^. Right ventricular blood pressure was measure by using a Millar Mikro-tip catheter transducer system as described^30^. Briefly, a Millar catheter (SPR-1000) was inserted into the right ventricle via external jugular vein cannulation. Blood pressure was recorded by using a LabVIEW software. Systolic ventricular blood pressure was measured and averaged from at least 10 cardiac cycles.

### Respiratory system compliance

An anesthetized mouse was placed in a supine position and a tracheostomy was performed with an 18-gauge needle. The intubated mouse was connected to a calibrated computer-controlled small animal ventilator FlexiVent (Scireq, Vancouver, Canada) and initially ventilated at a frequency of 150 breaths/min. (SnapShot-150), a tidal volume of 10 mL/kg, and PEEP of 2 cm H_2_O. After 5 minutes, respiratory system dynamic compliance was measured five times every 10 seconds at a tidal volume of 10, 15, 20, or 25 ml/kg sequentially using the FlexiVent SnapShot-150 perturbation. The snapshot was performed until three acceptable measurements were recorded in each animal, of which an average was calculated.

### Acute lung injury model

To induce lung injury, RenTgMK mice and WT littermates were treated with one dose (20 mg/kg) of lipopolysaccharide (LPS, O111:B4, Sigma L2630) by intratracheal instillation under anesthesia. After 24 hours, broncho-alveolar lavage (BAL) was collected, and protein concentration and cell number in the BAL fluid were measured. These data illuminate protein leak and cell infiltration from the circulation into the broncho-alveolar space. To directly measure pulmonary vascular leakiness, Evans blue (2%, MP Biomedicals) was delivered by retro-orbital injection 15 min before killing the mice. Evans blue that leaked into the lung interstitium was extracted and quantified by spectrophotometer as described previously^31^.

### Histology

Freshly harvested lungs were fixed in 10% formalin, processed and sectioned at 3 μm using a Leica Microtome. Sections were examined by routine H&E staining. Chord length and volume fraction of alveolar space, ductal air space and septal tissues were measured according to a published method^17^. In these volume fraction calculations, the percentage is calculated against the sum of the alveolar space, duct air space and septal tissue volume indirectly estimated based on point and intersection counting on histology images. Results were obtained from 10 randomly chosen microscopic fields in each mouse. Masson’s trichrome staining was used to assess fibrotic lesions in each section. ECM deposition areas were estimated using the computer software Image Pro Plus 6.0 (IPP6.0). Sections were also stained with anti-fibronectin (FN) antibodies. After incubation with horseradish peroxidase-conjugated secondary antibody, the slides were visualized with 3,3′-diaminobenzidine substrate (Sigma-Aldrich) and observed under a light microscope.

### RT-PCR

Total lung RNA was extracted using TRIzol reagent (Life Technologies). First-strand cDNAs were synthesized from total RNAs using MML-V reverse transcriptase (Life Technologies) and hexanucleotide random primers. Regular RT-PCR was performed using a BioRad S1000 Thermal Cycler. Real time PCR was carried out in a Roche 480 Real-Time PCR System, using SensiFAST SYBR No-Rox kits (Bioline). Beta-2 microglobulin served as an internal control. The amount of transcripts was calculated using the 2^−ΔΔCt^ formula. PCR primers are listed in Table 1.

### Western blot

Total lung lysates were prepared by homogenizing lung tissues in Laemmli buffer. Proteins were separated by SDS-PAGE and electroblotted onto Immobilon-P membranes. Western blotting analyses were carried out as previously described^32^. The antibodies used in this study included: TGF-β1, TGF-β3 (Abcam), fibronectin (Sigma-Aldrich) and α-smooth muscle actin (SMA) (Millipore). Secondary antibody was horseradish peroxidase-conjugated anti-IgG (Santa Cruz Biotechnology). Signals were visualized using SuperSignal West Dura Extended Duration Substrate (Pierce). The relative amount of proteins was quantified using gel analysis software UNSCAN-IT gel version 5.3 (Silk Scientific, Orem, UT), and normalized to β-actin internal loading control.

### Statistical analysis

Data values were presented as means ±SD. Statistical comparisons were carried out using unpaired two-tailed Student’s *t*-test for two group comparisons, and for three or more group comparisons two-way analysis of variance (ANOVA) with a Student-Newman-Keuls post-hoc test was used. P < 0.05 were considered statistically significant.

## Additional Information

**How to cite this article**: Wang, J. *et al.* Chronic Activation of the Renin-Angiotensin System Induces Lung Fibrosis. *Sci. Rep.* **5**, 15561; doi: 10.1038/srep15561 (2015).

## Supplementary Material

Supplementary Information

## Figures and Tables

**Table 1:** Nucleotide sequences of primers used in the study.

Gene name	Primer nucleotide sequence (5′–3′)
mFibronectin F	CGA GGT GAC AGA GAC CAC AA
mFibronectin R	CTG GAG TCA AGC CAG ACA CA
mTGF-b1 F	TGG AGC AAC ATG TGG AAC TCT
mTGF-b1 R	CCT GTA TTC CGT CTC CTT GGT
mCol-IVa F	AGG GTT ACC TGG AGA AAA AGG G
mCol-IVa R	TGG TCT CCT TTC TGT CCC TTC
mCTGF F	GTG TGC ACT GCC AAA GAT GGT G
mCTGF R	CAG CTT GAC CCT TCT CGG GAA
ma-SMA F	GAG GCA CCA CTG AAC CCT AA
ma-SMA R	CAT CTC CAG AGT CCA GCA CA
mCol-Ia F	GCA GGT TCA CCT ACT CTG TCC T
mCol-Ia R	CTT GCC CCA TTC ATT TGT CT
mCol-IIIa F	TCC CCT GGA ATC TGT GAA TC
mCol-IIIa R	TGA GTC GAA TTG GGG AGA AT
