# Supplementary material for: Chronic Activation of the Renin-Angiotensin System Induces Lung Fibrosis
Source: Sci Rep. 2015 Oct 23;5:15561. doi: 10.1038/srep15561 (PMC4616037; doi:10.1038/srep15561)
Supplement: Supplementary Information [file srep15561-s1.pdf]

Chronic Activation of the Renin-Angiotensin System Induces Lung Fibrosis

Jiaolong Wang, Li Chen, Bohao Chen, Angelo Meliton, Shu Q. Liu, Yongyan Shi, Tianjing Liu, Dilip K. Deb, Julian Solway, and Yan Chun Li

Fibronectin

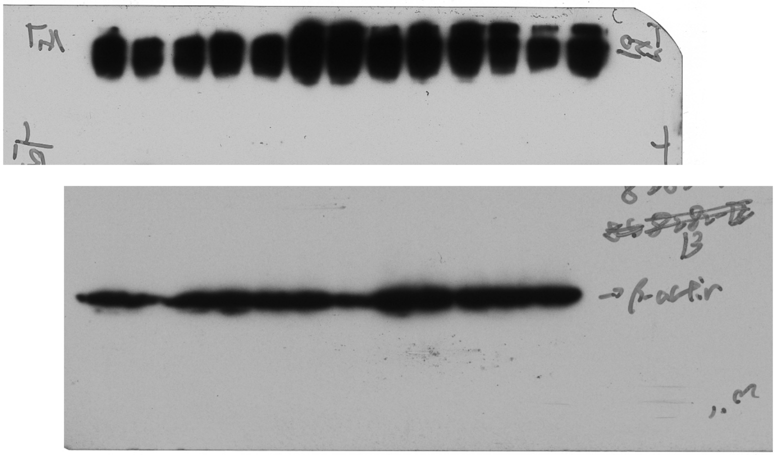

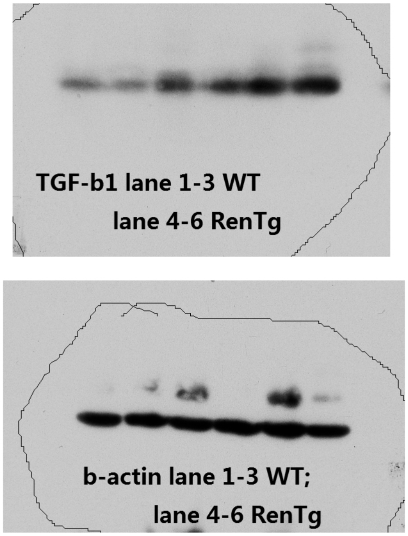

TGF-b1

TGF-b3

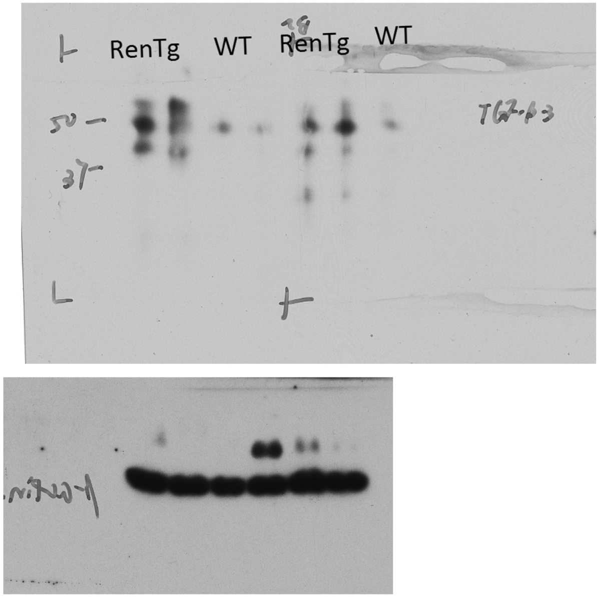

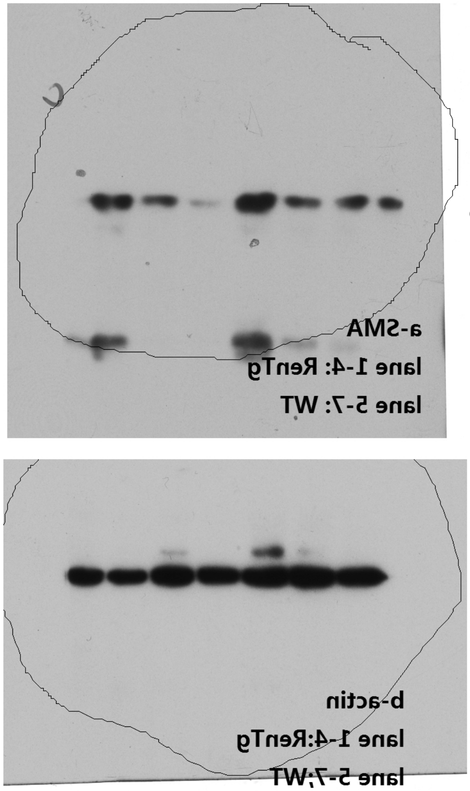

a-SMA

Figure 2B-E

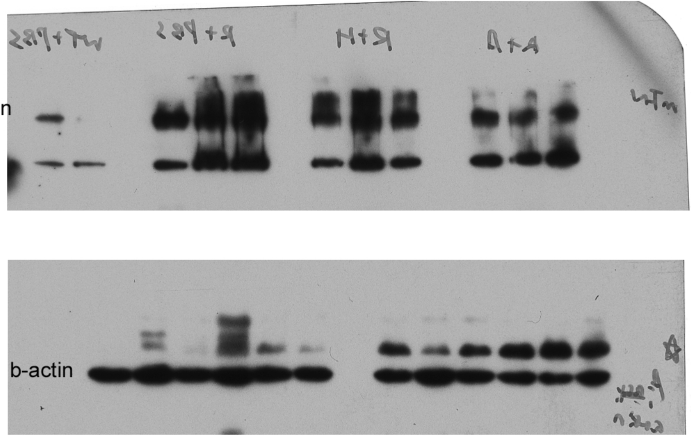

Figure 4B

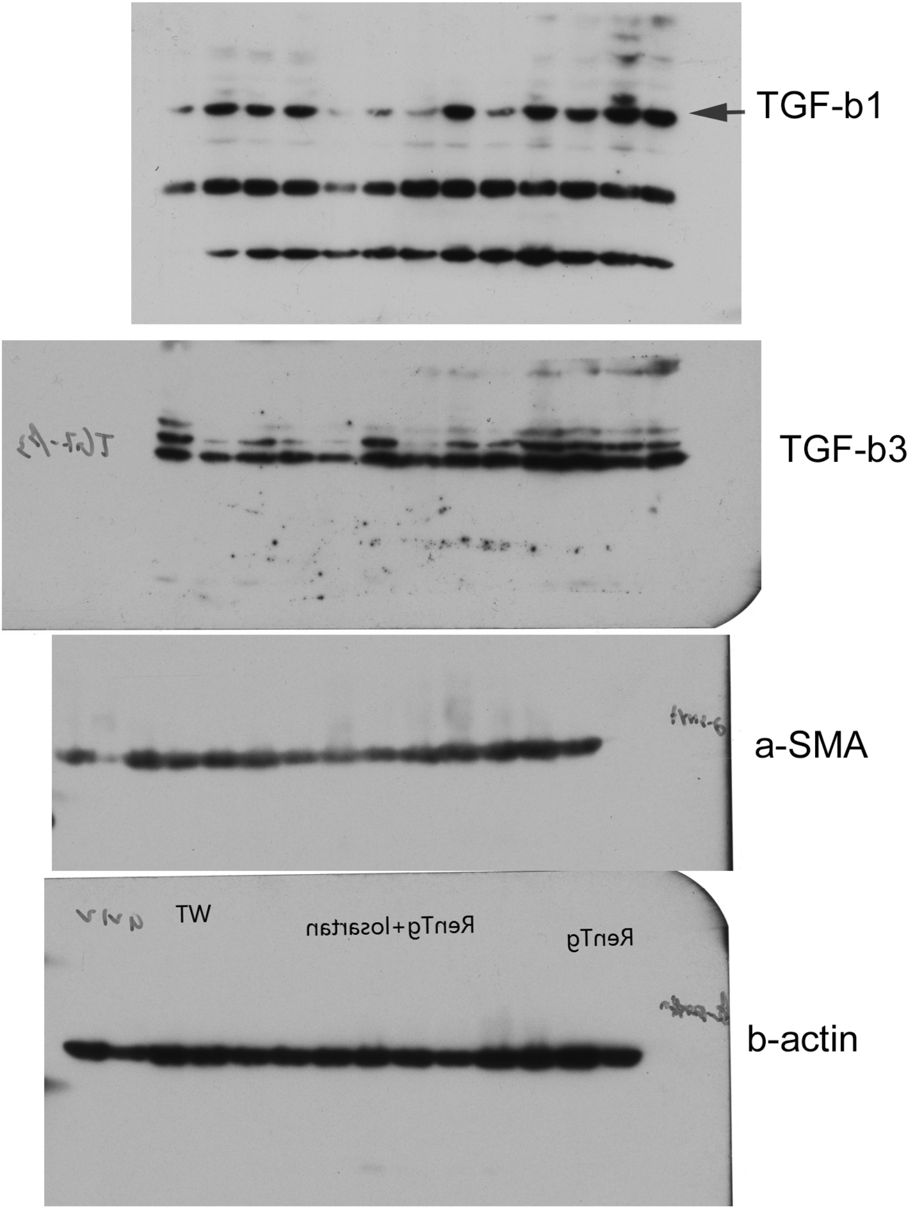

Figure 5E
